# Takotsubo Syndrome in Solid Organ Transplant Recipients: Current Evidence, Clinical Challenges, and Future Perspectives—A Narrative Review

**DOI:** 10.3390/jcm15208167

**Published:** 2026-10-20

**Authors:** Małgorzata Kosek-Nikołajczuk, Radosław Piątkowski, Marcin Grabowski, Monika Budnik

**Affiliations:** 1st Department of Cardiology, Medical University of Warsaw, Stefana Banacha 1a, 02-097 Warsaw, Poland

**Keywords:** Takotsubo syndrome, stress cardiomyopathy, solid organ transplantation, liver transplantation, catecholamines, cardiogenic shock, mechanical circulatory support

## Abstract

Takotsubo syndrome (TTS) is an acute and usually reversible form of myocardial dysfunction associated with emotional or physical stress. Solid organ transplantation (SOT) combines several potential triggers, including major surgical stress, hemodynamic instability, systemic inflammation, and exposure to exogenous catecholamines. Respiratory failure, calcineurin inhibitor toxicity, and altered cardiac innervation may also contribute. This narrative review summarizes current evidence on organ-specific timing, potential triggers, diagnostic challenges, management, and outcomes of TTS in SOT recipients. Reported cases predominantly occur early after liver and kidney transplantation, whereas presentations after heart transplantation range from the immediate postoperative period to years later. After heart transplantation, distinguishing TTS from early graft dysfunction and considering donor-side TTS are particularly important. Diagnostic certainty varies among published cases because formal criteria and the exclusion of competing diagnoses are inconsistently documented. Management largely follows recommendations for the general TTS population, with temporary mechanical circulatory support used in selected severe cases. Although ventricular function usually recovers, cardiogenic shock, arrhythmias, and multiorgan dysfunction may complicate the acute course, and persistent ventricular impairment has been associated with poorer long-term outcomes after liver transplantation. Comparisons with the general TTS population suggest a high proportion of severe presentations among reported transplant-associated cases; however, publication and ascertainment bias preclude firm conclusions about differences in incidence or severity. Evidence remains dominated by case reports, small series, and retrospective studies, underscoring the need for standardized reporting and prospective multicenter research.

## 1. Introduction

Takotsubo syndrome (TTS) is an acute and typically transient form of ventricular dysfunction, first described by Sato et al. in Japan in 1990 [1,2]. TTS accounts for approximately 1–3% of patients presenting with suspected acute coronary syndrome (ACS) [3]. Approximately 90% of cases occur in women, predominantly those older than 50 years [3]. The clinical presentation often resembles acute myocardial infarction (MI), with chest pain and dyspnea among the most common symptoms [4]. Several mechanisms have been implicated in its pathophysiology, including catecholamine-mediated myocardial injury, endothelial and microvascular dysfunction, oxidative stress, and abnormalities of coronary vasomotor regulation [3]. Both emotional and physical stress may precipitate TTS, with physical triggers reported slightly more frequently [4].

Surgery represents an important physical stressor, and perioperative TTS has been associated with the physiological burden of the procedure and marked sympathetic activation [5]. Solid organ transplantation (SOT) combines major surgical trauma with substantial hemodynamic and metabolic disturbances, inflammatory activation, and frequent perioperative exposure to vasoactive and inotropic agents. These factors provide a plausible setting for stress-induced myocardial dysfunction, while additional mechanisms may vary according to the transplanted organ. A scoping review by Rawashdeh et al. summarized TTS after solid organ transplantation up to 2023 [6]. Since then, multicenter cohort data [7] and several recent organ-specific reports have expanded the evidence base [8,9,10,11,12,13,14,15]. Our narrative review updates this literature and focuses on organ-specific differences in timing, triggers, diagnostic uncertainty, disease severity, management, prognosis, and current gaps in knowledge, while critically addressing the limitations of the available evidence.

### Literature Search Strategy

We conducted a non-systematic PubMed search for English-language publications addressing Takotsubo syndrome (TTS) in solid organ transplantation (SOT), including publications available up to 11 August 2026. The search covered liver, kidney, lung, and heart transplantation and included publications irrespective of year of publication. Particular attention was given to studies published after the period covered by the previous scoping review [6] in order to update the available evidence; however, earlier publications were retained when they represented relevant transplant-specific reports or provided important clinical or mechanistic context. The primary search combined the terms “Takotsubo syndrome”, “Takotsubo cardiomyopathy”, and “stress cardiomyopathy” with terms related to solid organ transplantation, including “solid organ transplantation”, “transplantation”, “liver transplantation”, “kidney transplantation”, “renal transplantation”, “lung transplantation”, and “heart transplantation”. The complete PubMed search strategy is provided in Supplementary Table S1. The primary search retrieved 88 records, all of which underwent title and abstract screening. Forty-one records were excluded at this stage, and 47 reports were assessed for relevance, of which 20 were retained in the core transplant-associated TTS narrative synthesis. Reference-list screening and targeted searches identified additional transplant-specific reports and were also used to support topics for which transplant-specific evidence was limited, including pathophysiology, diagnosis, and general TTS management. Consensus documents, reviews, studies from the general TTS population, and other relevant transplantation literature were used for contextual and supportive purposes. As this was a narrative review, no formal systematic-review protocol, PRISMA-based methodology, or structured risk-of-bias assessment was applied. The search was restricted to PubMed and English-language publications; therefore, relevant reports indexed exclusively in other databases or published in other languages may have been missed.

## 2. Why Are Transplant Recipients at Risk?

SOT is a physiologically demanding surgical procedure characterized by profound neuroendocrine activation, rapid hemodynamic fluctuations, systemic inflammation, and substantial perioperative emotional stress. Each of these factors may contribute to the development of TTS, suggesting that transplant recipients could represent a population particularly susceptible to stress-induced myocardial dysfunction [3,5,16]. Perioperative stress can activate the sympathetic nervous system and the hypothalamic–pituitary–adrenal axis, resulting in a rapid increase in circulating catecholamines and cortisol [1,3,16]. Excessive catecholamine exposure, including intravenous catecholamines administered perioperatively to treat vasoplegia or low cardiac output, may contribute to TTS by inducing direct myocardial toxicity, coronary microvascular dysfunction, calcium overload, and transient myocardial stunning [1,3].

The psychological burden associated with transplantation may also be relevant, as depressive symptoms are common among patients awaiting liver or kidney transplantation [17]. Emotional stress is a recognized trigger of TTS and may act alongside the substantial physical stress associated with transplantation [3,5].

Recently, Di Pietro et al. reported two cases of reversible cardiac failure in liver transplant recipients receiving tacrolimus [18]. Clinical evidence suggests that calcineurin inhibitors (CNIs), including cyclosporine A (CsA) and tacrolimus (TAC), which are widely used immunosuppressive agents in SOT recipients, may promote endothelial dysfunction, oxidative stress, mitochondrial injury, and impaired coronary microvascular function [19]. However, the extent to which these effects contribute specifically to SOT-related TTS remains unclear.

## 3. Organ-Specific Characteristics

### 3.1. Liver Transplantation

Reported estimates of TTS prevalence after liver transplantation (LT) vary considerably according to the method of case ascertainment. Zmaili et al., using multicenter pooled electronic health record data, identified 110 cases among 38,740 LT recipients (approximately 0.28%), whereas Yang et al., using a perioperative cohort, identified 38 cases among 2181 recipients (approximately 1.74%) [20,21]. More recently, Matar et al. reported 55 cases among 10,040 LT recipients (0.55%) in a multicenter cohort [7]. These differences likely reflect, at least in part, variation in surveillance and case detection rather than true biological differences in susceptibility [7,20,21]. Direct comparison with the general population is difficult because TTS rates after transplantation are reported within defined transplant cohorts, whereas general-population estimates are often based on patients presenting with suspected acute coronary syndrome [3,4,7,20,21]. Accordingly, the available data do not establish that TTS is intrinsically more prevalent after SOT. The greater number of published TTS cases after LT may reflect the substantial perioperative physiological stress of liver transplantation, as well as differences in surveillance, case ascertainment, and reporting [5,6].

Most LT-associated TTS develops early after transplantation, although intraoperative, delayed, and occasional pretransplant presentations have also been reported [7,20,22,23,24]. In the multicenter cohort by Matar et al., the median time to diagnosis was 4 days, with 69.1% of cases occurring within the first week. Median LVEF decreased from 64% before transplantation to 25% at diagnosis but recovered to ≥50% in 81.8% of patients. Failure of LVEF recovery was associated with significantly worse long-term survival [7].

Potential contributors to TTS after LT include substantial perioperative cardiovascular and hemodynamic stress associated with blood loss, transfusion requirements, vascular manipulation, and prolonged surgery [6,25], while cirrhosis-related alterations in the stress response may provide an additional substrate [6]. However, the contribution of individual intraoperative factors remains uncertain [7]. Comorbid cardiovascular disease, chronic kidney disease, sepsis, and psychological disorders may further increase susceptibility [6,21]. In the cohort reported by Yang et al., multivariable analysis identified alcohol-associated cirrhosis and higher MELD-Na scores as independent preoperative risk factors for TTS [20].

Despite its usually reversible nature, TTS after LT may have a severe clinical course. Bachayev et al. reported cardiogenic shock in approximately 32% of cases, followed by acute kidney injury (13%), arrhythmias (9%), and cardiac arrest (5%) [22]. In the EHR cohort, LT recipients with TTS more frequently required invasive mechanical ventilation and renal replacement therapy than LT recipients without TTS [21]. Severe cardiogenic shock may require temporary mechanical circulatory support, with successful use of intra-aortic balloon pumps (IABPs) and percutaneous ventricular assist devices reported after LT [25,26].

### 3.2. Kidney Transplantation

Similar to TTS after LT, most reported cases following KT occurred early after surgery, typically within the first 48 h [27,28,29]. Substantially fewer cases have been reported after KT than after LT [6]. However, this should not be interpreted as evidence of lower biological susceptibility, as differences in surveillance, case ascertainment, and reporting may also contribute. All five KT patients identified by Rawashdeh et al. developed cardiogenic shock with biventricular dysfunction and severely reduced LVEF [6]. In individual cases, tacrolimus toxicity and perioperative norepinephrine exposure have been proposed as potential contributing factors [28,29]. More recently, Eslami et al. described a 55-year-old woman who developed TTS within 12 h of KT, presenting with chest pain, dyspnea, diaphoresis, apical wall-motion abnormalities, and an LVEF of 25–30%, without significant coronary artery disease. Complete recovery of left ventricular (LV) systolic function was documented four weeks later [8]. Overall, evidence on TTS after KT remains limited to isolated case reports, making its incidence, risk factors, and outcomes difficult to determine.

### 3.3. Lung Transplantation

TTS in the setting of lung transplantation (LTx) has been reported across different peri-transplant and post-transplant time points and has been associated with severe respiratory and physiological stress. Michel-Cherqui et al. described TTS developing before LTx in a patient with end-stage lung disease and acute respiratory failure; urgent lung transplantation was subsequently performed 24 h later [30]. In contrast, Ghadri et al. reported atypical TTS seven years after bilateral LTx in association with acute respiratory distress syndrome and hypoxia [31]. Acute pulmonary disorders are also recognized triggers of TTS in the general population [32]. Hypoxia and hypercapnia may promote sympathetic activation through peripheral and central chemoreflex pathways, providing a plausible mechanistic link between respiratory failure and TTS [6,32]. Severe peri- and post-transplant cases have required extracorporeal support, including extracorporeal membrane oxygenation (ECMO) [9,13,33]. 

Diagnostic certainty varies among reported LTx-associated cases. In the case described by Boudart et al., persistent LV dysfunction at two-year follow-up and incomplete exclusion of perioperative coronary obstruction make the diagnosis less certain [13]. Similarly, in the case reported by Moradkhani et al., coronary angiography could not be performed because of the patient’s critical condition, and coronary CT angiography was not reported; although rapid recovery of LV function supported TTS, the absence of coronary imaging limits diagnostic certainty [9].

### 3.4. Heart Transplantation

In contrast to liver, kidney, and lung transplantation, several reported cases of TTS in heart transplant (HTx) recipients occurred months to years after transplantation and followed pharmacological stress testing, emotional stress, or other catecholaminergic stimuli [34,35,36]. Rare early presentations have also been described, including biventricular inverted TTS within the first day after HTx with inotropic support [37]. However, in this time window, distinction from early graft dysfunction (EGD) and other secondary causes of graft dysfunction is particularly important. Given the extrinsic cardiac denervation resulting from surgical disruption of sympathetic and parasympathetic nerve fibers, the occurrence of TTS after HTx is unexpected. However, variable degrees of sympathetic reinnervation have been documented [38,39,40]. Cardiac denervation and incomplete sympathetic reinnervation may alter adrenergic responsiveness after HTx, potentially increasing myocardial sensitivity to circulating catecholamines and susceptibility to TTS [38,39,40]. Furthermore, the absence of parasympathetic reinnervation may contribute to an exaggerated response to circulating catecholamines [39]. Recently, Covino et al. described emotionally triggered biventricular TTS in a 75-year-old woman 13 years after HTx. Despite cardiac denervation, the patient presented with chest pain and dyspnea, biventricular dysfunction (LVEF 38%), and a right ventricular thrombus. Coronary angiography showed no significant coronary artery disease, and ventricular function subsequently recovered completely [10].

According to the updated International Society for Heart and Lung Transplantation (ISHLT) consensus for heart transplantation, graft dysfunction of unknown cause occurring within the first 24 h after HTx is termed early graft dysfunction (EGD), whereas dysfunction occurring between 24 and 72 h is classified as subsequent graft dysfunction (sEGD) [41]. The updated classification further distinguishes immediate severe EGD-LV, characterized by failure to wean from cardiopulmonary bypass and the need for mechanical circulatory support, from delayed EGD-LV developing after successful separation from bypass [41]. Early ventricular dysfunction after HTx requires particular diagnostic caution because TTS may overlap clinically with EGD, acute rejection, and other causes of secondary graft dysfunction. TTS is suggested by a characteristic regional wall-motion pattern, often extending beyond a single coronary territory, together with subsequent recovery of ventricular function [3]. In practice, distinction from EGD should integrate the timing and severity of dysfunction, the need for mechanical circulatory support at or shortly after implantation, and donor and perioperative findings, while identifiable secondary causes should be actively excluded [41]. Further diagnostic testing should be guided by the suspected secondary cause; coronary assessment may be required when a coronary complication is suspected, while endomyocardial biopsy remains central to the evaluation of suspected acute rejection [42].

A related diagnostic consideration is donor-side TTS. Severe neurological injury and brain death may be accompanied by a marked catecholamine surge and transient myocardial dysfunction resembling stress cardiomyopathy [43]. Consequently, TTS may already be present in the donor heart before transplantation [43,44]. This possibility has also been suggested in individual reports, including the case described by Yasumura et al. [15]. More recently, Giovannico et al. reported seven heart transplantations from donors with echocardiographic evidence of TTS at the time of brain death and full or partial recovery before procurement. Obstructive coronary disease was excluded by angiography; all recipients were alive at 12 months, and mean LVEF increased from 52 ± 6% at 1 week after transplantation to 58 ± 4% at 12 months, with progressive improvement in GLS during follow-up [44]. Review of donor echocardiography and the trajectory of ventricular recovery before procurement may therefore help distinguish pre-existing donor myocardial stunning from de novo recipient TTS or early graft dysfunction. These observations also suggest that selected donor hearts with recovered TTS may be suitable for transplantation [44].

### 3.5. Multivisceral Transplantation

Souki et al. reported the first case of TTS following multivisceral transplantation (MVT) in a 61-year-old man with alcohol-associated cirrhosis and extensive mesenteric thrombosis. TTS developed on the second postoperative day, with an LVEF of 35–40%, apical and mid-ventricular hypokinesis, QTc prolongation, and elevated cardiac biomarkers. Potential triggers included prolonged surgery (>8 h), massive intraoperative hemorrhage (24 L), extensive transfusion, large-volume fluid administration, and exposure to multiple vasopressors and epinephrine. The patient received intraoperative venovenous ECMO support and later developed atrial fibrillation and required reintubation. Cardiac function recovered completely by postoperative day 23, with an LVEF of 55–60% [11].

**Figure 1:**
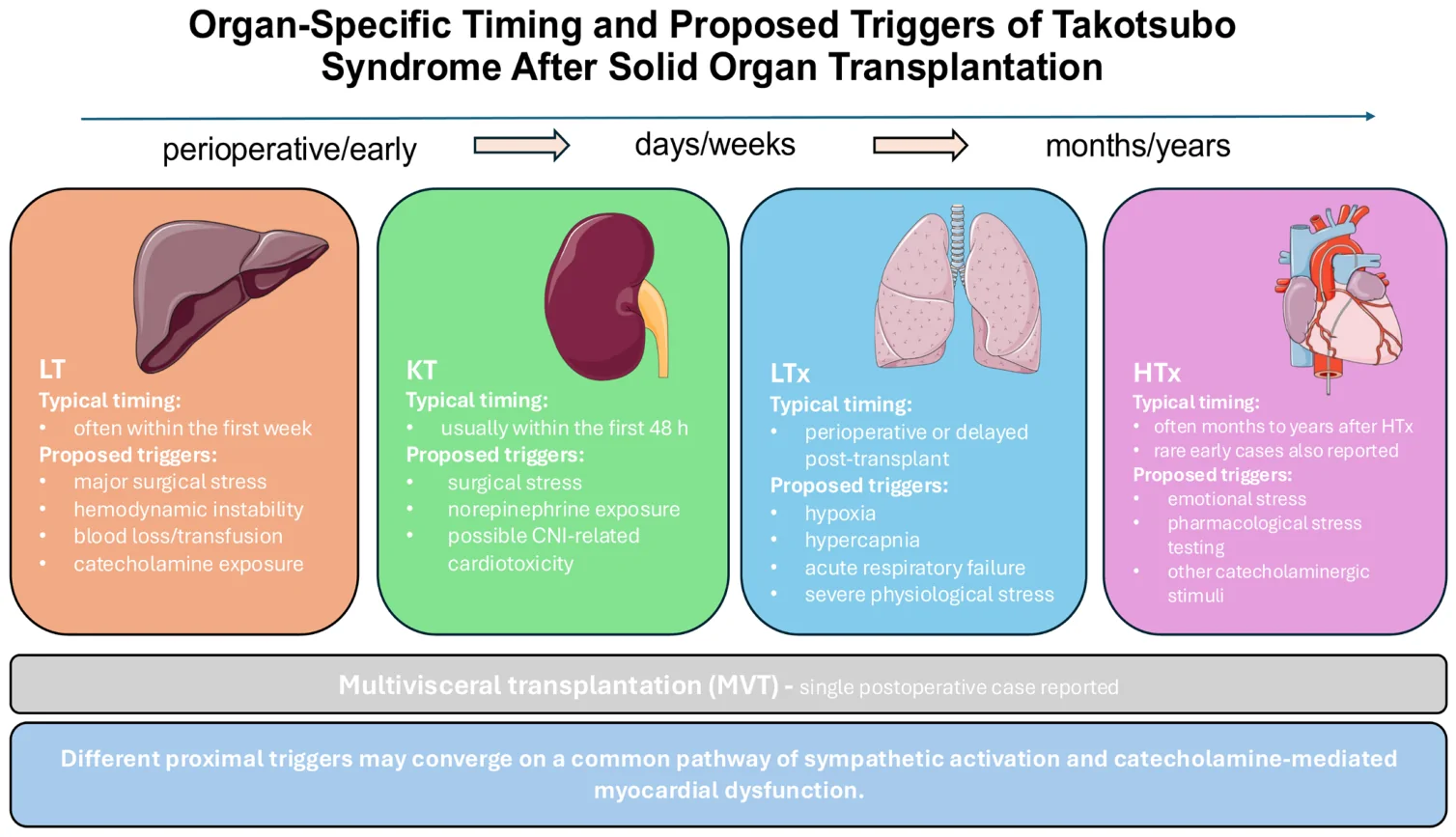
Organ-specific timing and proposed triggers of Takotsubo syndrome after solid organ transplantation. Abbreviations: CNI, calcineurin inhibitor; HTx, heart transplantation; KT, kidney transplantation; LT, liver transplantation; LTx, lung transplantation; MVT, multivisceral transplantation; SOT, solid organ transplantation; TTS, Takotsubo syndrome. Images adapted from Servier Medical Art (https://smart.servier.com/, accessed on 2 October 2026), licensed under CC BY 4.0 (https://creativecommons.org/licenses/by/4.0/).

Although early and delayed TTS after transplantation arise in different clinical settings, they may converge on a common final pathway of excessive sympathetic activation and catecholamine-mediated myocardial dysfunction [3,6]. Early perioperative cases are more often associated with major surgery, hemodynamic instability, blood loss, respiratory failure, and exogenous catecholamine exposure, whereas delayed cases may follow emotional stress, seizures, diagnostic or pharmacological stress testing, or other catecholaminergic stimuli [6,10]. After HTx, cardiac denervation and variable sympathetic reinnervation may further modify myocardial responsiveness to circulating catecholamines [38,39,40]. Table 1 summarizes selected recent studies and case reports illustrating these organ-specific differences. Organ-specific timing and proposed triggers are summarized in Figure 1.

## 4. Clinical Presentation

The clinical presentation of TTS after SOT generally resembles that observed in the non-transplant population, although symptoms may vary according to the transplanted organ. Dyspnea and chest discomfort are among the more commonly reported symptoms, whereas dizziness, generalized weakness, and syncope have been described less consistently [1,3,6]. In KT recipients, reported presentations have included chest pain, dyspnea, diaphoresis, ECG abnormalities, and elevated troponin levels [8,27,28,29]. In reported LTx cases, dyspnea and respiratory deterioration have been prominent, particularly when TTS occurred in the setting of acute respiratory failure or hypoxemia [9,13,31]. In several reported HTx cases, chest pain was absent, and patients presented predominantly with heart failure symptoms such as acute dyspnea and desaturation [10,34,37,38]. This may be related to cardiac denervation and incomplete neural reinnervation after HTx [38,39,40]. Because of its overlap with ACS and other causes of acute cardiac or graft dysfunction, TTS may be difficult to recognize after SOT, particularly when typical symptoms such as chest pain are absent.

## 5. Diagnostic Challenges

The diagnosis of TTS in SOT recipients may be particularly challenging because its clinical presentation, electrocardiographic abnormalities, and elevated cardiac biomarkers frequently mimic ACS [6,48]. Moreover, transplant recipients are susceptible to several alternative causes of acute cardiac dysfunction, including perioperative myocardial injury, sepsis-related cardiomyopathy, myocarditis, hemodynamic instability, and drug-related cardiotoxicity [6]. In HTx recipients, acute rejection and cardiac allograft vasculopathy should also be considered in the differential diagnosis [6,10,34]. The absence of typical symptoms may further complicate recognition, particularly after HTx, where chest pain may be absent because of cardiac denervation [38,39,40]. Major organ-specific competing diagnoses are summarized in Table 2.

The diagnosis of TTS is based on criteria used in the non-transplant population, including the 2018 InterTAK Diagnostic Criteria and the modified Mayo Clinic criteria [3,48,49]. Echocardiography is the most widely used imaging modality and allows the detection of regional wall-motion abnormalities that typically extend beyond a single epicardial coronary vascular territory, as well as atypical patterns and right ventricular involvement [48]. Because TTS frequently mimics ACS, several clinical and laboratory parameters have been investigated as potential diagnostic clues to distinguish TTS from STEMI [50]. However, coronary angiography is frequently required to exclude an acute coronary culprit lesion, particularly in patients presenting with ST-segment elevation or other features suggestive of ACS [48,49]. Cardiac magnetic resonance (CMR) provides high-resolution tissue characterization and may be particularly valuable in uncertain cases, helping to differentiate TTS from myocardial infarction and myocarditis [48,49].

Serial echocardiographic assessment is important because recovery of ventricular function supports the transient nature of myocardial dysfunction and provides prognostic information [7,48]. The presence of significant coronary artery disease does not exclude TTS, as the two conditions may coexist [3,48,49]. Atypical morphological variants, right ventricular involvement, and biventricular TTS may further complicate diagnosis [10,48]. TTS should therefore be considered in SOT recipients who develop otherwise unexplained acute ventricular dysfunction, particularly following major surgery, catecholamine exposure, or severe physiological or emotional stress [6].

**Figure 2:**
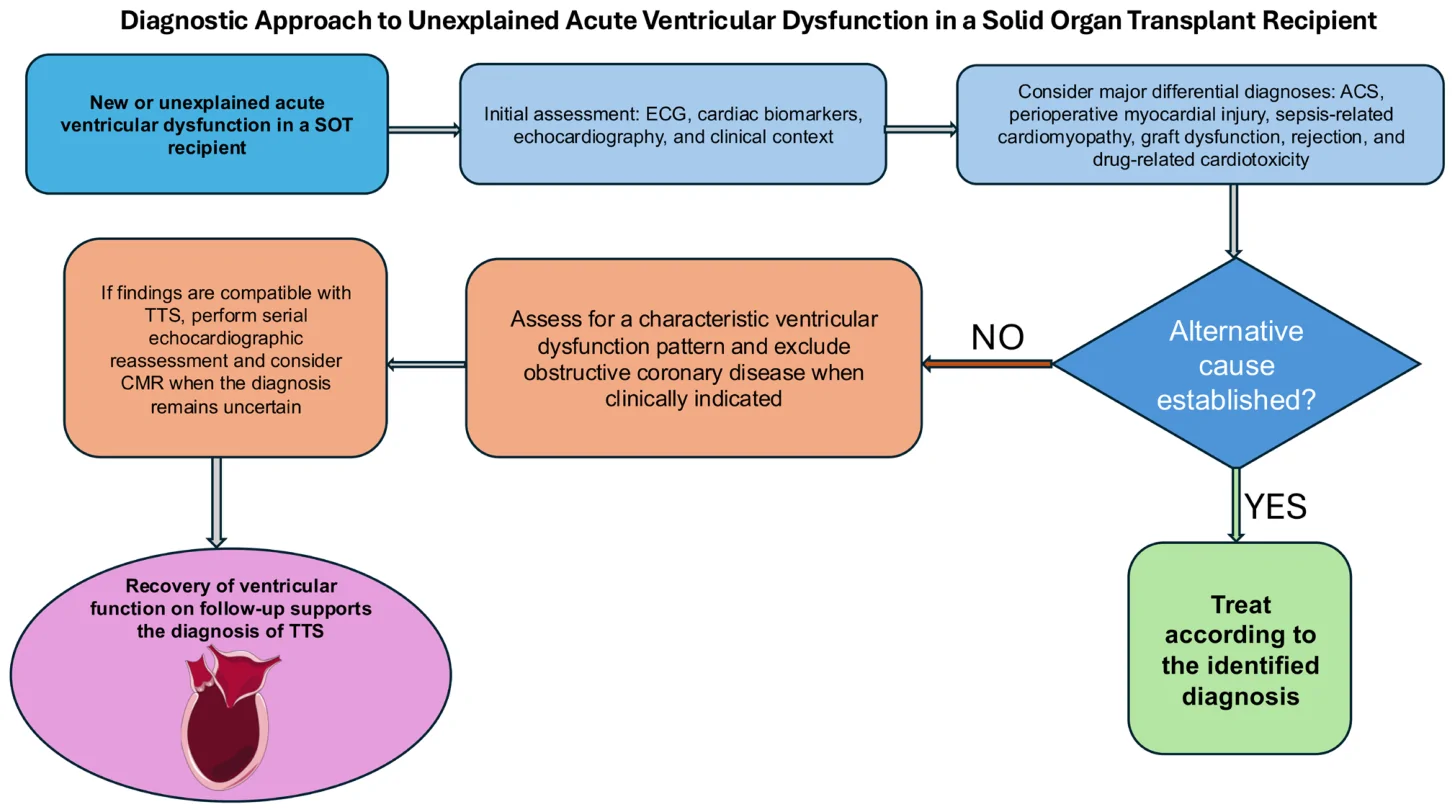
Diagnostic approach to unexplained acute ventricular dysfunction in a solid organ transplant recipient. The algorithm summarizes a practical stepwise approach including initial clinical, electrocardiographic, biomarker, and echocardiographic assessment; evaluation for major alternative diagnoses; assessment for a characteristic ventricular dysfunction pattern; exclusion of obstructive coronary disease when clinically indicated; and serial reassessment with echocardiography and, when needed, cardiac magnetic resonance. Recovery of ventricular function during follow-up provides additional diagnostic support for TTS. Abbreviations: ACS, acute coronary syndrome; CMR, cardiac magnetic resonance; ECG, electrocardiography; SOT, solid organ transplantation; TTS, Takotsubo syndrome. Heart illustration adapted from Servier Medical Art (https://smart.servier.com/, accessed on 2 October 2026), licensed under CC BY 4.0 (https://creativecommons.org/licenses/by/4.0/).

An additional challenge is the variable diagnostic certainty of published transplant-associated TTS cases. Several published reports predate current diagnostic frameworks [3,48,49] or do not explicitly state whether formal InterTAK or Mayo criteria were applied [9,22,46,47]. In some cases, coronary imaging or cardiac magnetic resonance was not reported, making exclusion of alternative causes of acute ventricular dysfunction difficult [9,13,46,47]. This is particularly relevant in SOT recipients, in whom perioperative myocardial injury, sepsis-related cardiomyopathy, graft dysfunction, rejection, and drug-related cardiotoxicity may produce overlapping clinical and imaging findings, depending on the transplanted organ [6,41,42]. Accordingly, reported cases should not necessarily be regarded as diagnostically equivalent, and the available literature should be interpreted in light of the completeness of the diagnostic work-up. A practical diagnostic approach is outlined in Figure 2.

## 6. Treatment and Prognosis

There are currently no specific evidence-based recommendations for the treatment of TTS in SOT recipients. Management is largely extrapolated from recommendations for TTS in the general population and adapted to the individual hemodynamic profile [6,48]. In hemodynamically stable patients, treatment is primarily supportive, with heart failure therapy, including diuretics and, when appropriate, afterload reduction, guided by clinical presentation [48,49]. Concomitant conditions, including infection, respiratory failure, and other sources of substantial physiological stress, should be identified and treated [6,49]. The potential contribution of exogenous catecholamines and CNIs should also be considered, although evidence for a direct causal role remains limited [6].

During the acute phase, ECG monitoring is essential to detect QT prolongation and potentially life-threatening ventricular arrhythmias; QT-prolonging medications should be avoided where possible [48]. Anticoagulation should be considered in patients with severe LV dysfunction and extensive apical ballooning because of the risk of ventricular thrombus and embolism [48,49]. In transplant recipients, this decision requires individualized assessment of thromboembolic and bleeding risks, particularly in the early postoperative period.

Reported TTS after SOT appears to be characterized by a relatively high proportion of severe presentations. In the International Takotsubo Registry, cardiogenic shock occurred in 8.3% of 3740 patients with TTS, whereas Bachayev et al. reported cardiogenic shock in 32% of published LT cases, and all five KT cases identified in the scoping review by Rawashdeh et al. were complicated by cardiogenic shock [6,22,51]. In the general TTS population, left ventricular outflow tract obstruction (LVOTO) has been reported in approximately 7–25% of cases [51], right ventricular involvement in approximately 11% [52], and 112 of 4679 patients (2.4%) included in a meta-analysis died during the index hospitalization [53]. Comparable transplant-specific estimates for LVOTO, mortality, and atypical or biventricular variants are not currently available [6,22]. These comparisons should be interpreted cautiously because transplant-specific evidence is dominated by case reports, small series, and retrospective studies, with substantial potential for publication and ascertainment bias [6,22]. Consequently, the high proportion of severe presentations among reported SOT-associated TTS cases may not reflect the true distribution of disease severity in the transplant population. The available evidence therefore does not establish that TTS after SOT is intrinsically more severe than TTS in the general population.

Cardiogenic shock, as one of the most severe complications of TTS, requires careful assessment of its underlying mechanism, particularly the presence of LVOTO, which substantially influences therapeutic decisions [6,48]. Catecholamines should be administered cautiously because excessive adrenergic stimulation is implicated in the pathophysiology of TTS; however, vasopressors or inotropes may be unavoidable in critically ill transplant recipients [6,48]. In refractory cardiogenic shock, temporary mechanical circulatory support may provide a bridge to myocardial recovery [54]. Recent data from the International Takotsubo Registry provide additional information on device selection. In this registry analysis, after propensity-score matching, IABP use was associated with lower in-hospital mortality than medical therapy alone (14.5% vs. 35.5%, *p* = 0.002), whereas in-hospital mortality did not differ significantly between Impella and medical therapy (25.0% vs. 29.2%, *p* = 0.75). Device-related complications occurred in 6.0% of patients treated with IABP and 31.3% of those treated with Impella [51]. These observational findings should not be interpreted as establishing superiority of one device and emphasize individualized device selection according to the underlying hemodynamic mechanism and local expertise [51]. The presence of LVOTO should be carefully assessed because it substantially influences management [48]. Importantly, IABP should be avoided in the presence of significant LVOTO, as it may aggravate the outflow gradient [51]. In SOT recipients, IABP, percutaneous ventricular assist devices, and ECMO have all been used in patients with severe hemodynamic compromise [6,9,13,25]. Available data do not permit a reliable comparison of MCS utilization between SOT recipients and the general TTS population [6,22,51,54].

Although TTS is characterized by reversible myocardial dysfunction, increasing evidence from the general TTS population indicates that its long-term prognosis is not necessarily benign [55]. This also appears to apply to SOT recipients, in whom severe acute complications and adverse long-term outcomes have been reported [6,7]. Cardiogenic shock, arrhythmias, cardiac arrest, acute kidney injury, and the need for mechanical ventilation or renal replacement therapy have all been reported in SOT recipients with TTS [6,21,22]. Nevertheless, ventricular function recovers in the majority of survivors [6,7]. In the multicenter cohort study by Matar et al., LVEF recovered to ≥50% in 81.8% of liver transplant recipients, despite a decline in median LVEF from 64% before transplantation to 25% at TTS diagnosis. Patients without LVEF recovery had substantially worse long-term survival, highlighting the prognostic importance of follow-up echocardiography. Failure of ventricular recovery may therefore have prognostic significance in LT recipients [7].

## 7. Knowledge Gaps and Future Directions

Evidence on TTS in SOT recipients is scarce and consists predominantly of individual case reports, small case series, retrospective analyses, and literature reviews. Larger observational cohort studies have been conducted almost exclusively in LT recipients [7,20,21]. Consequently, the incidence of TTS after KT, LTx, HTx, and MVT remains unknown. The predominance of reported cases after LT may reflect true differences in susceptibility as well as differences in recognition and reporting. Moreover, preferential publication of severe or unusual cases may affect the reported spectrum of clinical presentations and outcomes. Differences in reported incidence across transplant types and individual studies should therefore be interpreted cautiously, as they may reflect variation in surveillance intensity, diagnostic work-up, and case ascertainment rather than true biological differences in susceptibility.

The contribution of individual transplant-related factors to TTS is also unclear. Perioperative catecholamine exposure, hemodynamic instability, hypoxia, systemic inflammation, and CNI-related cardiotoxicity have all been proposed as potential contributors. However, the available data make it difficult to determine their individual contributions and potential interactions [6,7,19,20]. The occurrence of TTS in a denervated transplanted heart is particularly noteworthy, as it suggests a complex relationship between circulating catecholamines, adrenergic receptor sensitivity, and variable sympathetic reinnervation [37,38,39,40]. It is also unknown whether recipient characteristics, underlying organ disease, immunosuppressive regimens, or specific aspects of perioperative management influence TTS risk.

No diagnostic or therapeutic recommendations have been developed specifically for transplant recipients, and current practice is based largely on evidence from the general TTS population [48,49]. Prospective multicenter studies and transplant registries could provide more reliable estimates of organ-specific incidence and help identify factors associated with TTS and its outcomes. Consistent reporting of potential triggers, catecholamine exposure, ventricular phenotype, biomarkers, imaging findings, immunosuppressive therapy, mechanical circulatory support, and ventricular recovery would also improve comparisons across studies. A suggested minimum reporting dataset is provided in Box 1. Such data could help determine which transplant recipients are at greatest risk and whether modifications to perioperative management or immunosuppressive therapy could reduce that risk.

Box 1 **Suggested minimum reporting dataset for TTS after solid organ transplantation.**

- Transplanted organ and indication for transplantation
- Timing of TTS relative to transplantation
- Potential physical/emotional trigger
- Perioperative catecholamine and vasopressor exposure
- Immunosuppressive regimen
- ECG and cardiac biomarkers
- Echocardiographic phenotype and LVEF
- RV involvement and LVOTO
- Coronary assessment
- CMR, if performed
- Diagnostic criteria used
- MCS requirement
- Time to ventricular recovery
- Short- and long-term outcomes

## 8. Conclusions

Takotsubo syndrome is a potentially serious cardiovascular complication of solid organ transplantation, with organ-specific differences in timing, triggers, and diagnostic challenges. It should be considered in transplant recipients with otherwise unexplained acute ventricular dysfunction, particularly following major perioperative stress or catecholamine exposure. Because TTS may mimic ACS, perioperative myocardial injury, sepsis-related cardiomyopathy, drug-related cardiotoxicity, and, after heart transplantation, EGD or rejection, diagnosis should be based on appropriate exclusion of alternative causes and recognition of a characteristic ventricular dysfunction pattern, with subsequent recovery providing additional diagnostic support. Although ventricular function recovers in most reported cases, severe complications, including cardiogenic shock and multiorgan dysfunction, may occur. Future multicenter studies and standardized reporting are needed to define organ-specific incidence, risk factors, diagnostic pathways, and management strategies.

## Figures and Tables

**Table 1:** Selected recent studies and reports of TTS after solid organ transplantation (2021–2026).

Diagnostic Assessment	Outcome	Main Clinical Features	Proposed Trigger(s)/Associations	Time to TTS	No. of Patients	Type of Study	Reference	Organ
2018 consensus criteria used; individual coronary imaging and CMR were not reported for all cases	Full cardiac recovery in all TTS patients; 1-year survival comparable with controls	Acute LV systolic dysfunction; mean LVEF 25.5% ± 7.8%	Alcohol-associated cirrhosis and higher MELD-Na: independent preoperative risk factors; higher epinephrine and lower fentanyl doses associated with TTS after PSM	71% within 5 days	38 TTS/2181 LT	Retrospective cohort with PSM	Yang et al., 2021 [20]	Liver
EHR-based case identification; individual diagnostic work-up, coronary imaging and CMR unavailable	Higher in-hospital morbidity; mortality not reported	Cardiogenic shock/cardiac arrest; increased need for ventilation and RRT	Older age, female sex, Caucasian ethnicity, and arrhythmias associated with TTS; cardiovascular/systemic comorbidities common	Cumulative diagnoses plateaued at ~16 weeks	110 TTS/38,740 LT	Multicenter pooled EHR cohort	Zmaili et al., 2022 [21]	Liver
Apical ballooning; InterTAK score 73; authors reported Mayo criteria fulfilled; no CAD on angiography; CMR not reported	Recovery of LV function by POD 20	Apical ballooning; LVEF ~35%; no obstructive CAD	Major perioperative stress	POD 6	1	Case report	Badwan et al., 2023 [45]	Liver
Heterogeneous diagnostic work-up; formal criteria and coronary/CMR imaging varied across reports	MCS required in 30.3%; recurrence infrequently reported; mortality 30.4%	Hypotension 30%; dyspnea 14%; apical ballooning 46.5%; cardiogenic shock 32%	Perioperative/surgical stress; catecholaminergic mechanisms	82% postoperative; 14% intraoperative	56 cases *	Systematic review and pooled analysis	Bachayev et al., 2024 [22]	Liver
Reverse TTS on intraoperative TEE; no formal criteria, coronary angiography, or CMR reported; LVEF recovered by POD 10	LVEF 50% by POD 10; no further instability	Reverse TTS with basal hypokinesia and apical sparing; LVEF 20%; ventricular tachycardia	Major surgical/hemodynamic stress	Intraoperative	1	Case report	Pal et al., 2024 [46]	Liver
Typical apical ballooning on CTA/echo; preoperative angiography showed no obstructive CAD; formal criteria and CMR not reported	Complete recovery of cardiac function	Apical LV dilatation on CTA; apical ballooning and severe mid-apical hypokinesis; LVEF 25%; elevated troponin	Not clearly established	4 weeks after LT	1	Imaging report	Jehangir, 2025 [47]	Liver
TTS classified primarily by apical ballooning on echocardiography/cardiac catheterization; coronary angiography was not systematically performed, and ACS could not be excluded in one patient; CMR not reported	5 patients survived with cardiac recovery; 1 died of acute cardiac failure	Acute LV dysfunction; cardiogenic shock/hemodynamic instability in 5 of 6 patients (83%)	Perioperative hemodynamic stress; catecholamine excess documented in 1 case	Median 2 days after LT	6 TTS/739 LT	Case series	Zhao et al., 2025 [12]	Liver
Mayo Clinic criteria used; all patients underwent pre- and post-LT echocardiography; post-LT coronary angiography was performed in 19/55 and CCTA in 1/55; follow-up echocardiography documented recovery in most patients	LVEF recovered to ≥50% in 81.8%; lack of recovery associated with markedly worse long-term survival	Median LVEF 64% → 25%; global hypokinesis 41.8%; apical involvement in 81.3% of regional variants	No specific trigger identified; contribution of perioperative factors uncertain.	Median 4 days (range 1–74); 69.1% <7 days	55 TTS/10,040 LT	Multicenter retrospective cohort	Matar et al., 2025 [7]	Liver
Apical ballooning with severe LV dysfunction; no significant CAD on angiography; complete recovery at 4 weeks; CMR not reported	Complete normalization of LV function at 4 weeks	Chest pain, dyspnea, diaphoresis; ST–T changes; elevated troponin; apical WMA; LVEF 25–30%; unobstructed coronaries	Postoperative physiological stress; norepinephrine exposure.	Within 12 h after KT	1	Case report	Eslami et al., 2025 [8]	Kidney
HFA criteria reportedly met; preoperative angiography normal, no repeat angiography during event; persistent LV dysfunction and incomplete exclusion of coronary obstruction reduce certainty; CMR not reported	VA-ECMO; persistent LV dysfunction at 2 years	Severe LV failure, ST-segment elevation, diffuse hypokinesia, cardiogenic shock and multiorgan dysfunction	Major surgical/physiological stress; severe cardiopulmonary instability	Intraoperative during bilateral LTx	1	Case report	Boudart et al., 2024 [13]	Lung
No formal criteria stated; coronary angiography could not be performed because of critical illness; CCTA not reported; ACS considered less likely; rapid LV recovery supported TTS	ECMO and supportive therapy; recovery within 3 weeks; later LV dysfunction lacked typical features of recurrent TTS	Acute TTS with reduced LVEF	Major surgical/physiological stress associated with LTx.	Intraoperative during bilateral LTx	1	Case report	Moradkhani et al., 2026 [9]	Lung
Consensus criteria used; angiography excluded obstructive CAD; biopsy excluded rejection; wall motion normalized by day 19; CMR not reported	LV wall motion normalized by day 19	Apical ballooning after seizure; ^123^I-MIBG demonstrated partial sympathetic reinnervation	Status epilepticus; partial sympathetic reinnervation	15 months after HTx	1	Case report	Shikama et al., 2025 [14]	Heart
Biventricular TTS supported by angiography showing no significant coronary stenosis and CMR without LGE; RV apical thrombus; complete recovery; formal criteria not explicitly stated	LVEF 52% at discharge and 55% at follow-up; complete resolution of RV thrombus	Chest pain and dyspnea; biventricular TTS; LVEF 38%; RV thrombus; no significant CAD	Emotional stress	13 years after HTx	1	Case report	Covino et al., 2025 [10]	Heart
Recurrent transient apical/biventricular TTS with rapid recovery; coronary CT/angiography and endomyocardial biopsy excluded major alternative causes; ^123I-MIBG showed minimal myocardial uptake, indicating limited sympathetic reinnervation	Wall-motion abnormalities normalized within days after both post-HTx episodes	Recurrent biventricular/apical TTS; ^123I-MIBG showed minimal myocardial uptake	TTS despite limited sympathetic reinnervation; donor heart with probable TTS	4 and ~5 years after HTx	1	Case report	Yasumura et al., 2025 [15]	Heart
Apical ballooning; InterTAK score 55; complete recovery by POD 23; coronary angiography and CMR not reported	Complete recovery, LVEF 55–60% by POD 23	LVEF 35–40%; apical/mid-ventricular hypokinesis; QTc prolongation	Prolonged surgery, massive blood loss/transfusion, fluid shifts, multiple vasopressors including epinephrine	POD 2	1	Case report	Souki et al., 2025 [11]	Multivisceral

Diagnostic assessment reflects the information reported in the original publications. “Not stated” or “not reported” indicates that the use of formal diagnostic criteria or a specific imaging modality was not explicitly described and does not necessarily imply that it was not performed. * The systematic review included 56 case reports from 30 articles and additionally analyzed 10 original studies. Abbreviations: TTS, Takotsubo syndrome; LT, liver transplantation; KT, kidney transplantation; LTx, lung transplantation; HTx, heart transplantation; LVEF, left ventricular ejection fraction; LV, left ventricle; RV, right ventricle; WMA, wall-motion abnormalities; CAD, coronary artery disease; CKD, chronic kidney disease; POD, postoperative day; RRT, renal replacement therapy; MCS, mechanical circulatory support; ECMO, extracorporeal membrane oxygenation; VA-ECMO, veno-arterial extracorporeal membrane oxygenation; CTA, computed tomography angiography; MIBG, meta-iodobenzylguanidine; MELD-Na, Model for End-Stage Liver Disease—Sodium; EHR, electronic health record; ACS, acute coronary syndrome; CCTA, coronary computed tomography angiography; CMR, cardiac magnetic resonance; HFA, Heart Failure Association; PSM, propensity-score matching; TEE, transesophageal echocardiography; LGE, late gadolinium enhancement.

**Table 2:** Major competing diagnoses in suspected TTS after solid organ transplantation.

Important Competing Diagnoses	Organ
Cirrhotic cardiomyopathy; post-reperfusion syndrome; ACS/perioperative myocardial injury; sepsis-related cardiomyopathy; transfusion-related acute lung injury (TRALI)/transfusion-associated circulatory overload (TACO)	LT
ACS/perioperative myocardial injury; volume overload; uraemic cardiomyopathy; CNI-related cardiotoxicity; high-output heart failure related to arteriovenous fistula	KT
Primary graft dysfunction (PGD), including severe PGD grade 3; RV failure; pulmonary embolism; ACS/perioperative myocardial injury; hypoxia-related myocardial dysfunction; sepsis-related cardiomyopathy	LTx
Early graft dysfunction (EGD); subsequent graft dysfunction (sEGD); acute rejection; cardiac allograft vasculopathy; coronary or surgical complications	HTx

Abbreviations: ACS, acute coronary syndrome; CNI, calcineurin inhibitor; EGD, early graft dysfunction; HTx, heart transplantation; KT, kidney transplantation; LT, liver transplantation; LTx, lung transplantation; PGD, primary graft dysfunction; RV, right ventricular; sEGD, subsequent graft dysfunction; TACO, transfusion-associated circulatory overload; TRALI, transfusion-related acute lung injury.

## Data Availability

No new data were created or analyzed in this study. Data sharing is not applicable to this article.

## Supplementary Materials

The following supporting information can be downloaded at https://www.mdpi.com/article/10.3390/jcm15208167/s1, Supplementary Table S1. PubMed search string.

## References

[1] Lyon A.R., Bossone E., Schneider B., Sechtem U., Citro R., Underwood S.R., Sheppard M.N., Figtree G.A., Parodi G., Akashi Y.J. (2016). Current state of knowledge on Takotsubo syndrome: A Position Statement from the Taskforce on Takotsubo Syndrome of the Heart Failure Association of the European Society of Cardiology. Eur. J. Heart Fail..

[2] Sato H., Tateishi H., Uchida T., Kodama K., Haze K., Hon M. (1990). Takotsubo-type cardiomyopathy due to multivessel spasm. Clinical Aspect of Myocardial Injury: From Ischemia to Heart Failure.

[3] Ghadri J.R., Wittstein I.S., Prasad A., Sharkey S., Dote K., Akashi Y.J., Cammann V.L., Crea F., Galiuto L., Desmet W. (2018). International Expert Consensus Document on Takotsubo Syndrome (Part I): Clinical Characteristics, Diagnostic Criteria, and Pathophysiology. Eur. Heart J..

[4] Templin C., Ghadri J.R., Diekmann J., Napp L.C., Bataiosu D.R., Jaguszewski M., Cammann V.L., Sarcon A., Geyer V., Neumann C.A. (2015). Clinical Features and Outcomes of Takotsubo (Stress) Cardiomyopathy. N. Engl. J. Med..

[5] Shang Z., Zhao M., Cai J., Wu C., Xu Y., Zeng L., Cai H., Xu M., Fan Y., Li Y. (2022). Peri-operative Takotsubo syndrome after non-cardiac surgery: A retrospective nested case-control study. ESC Heart Fail..

[6] Rawashdeh B., Yaghmour N., Sulieman D., Abuassi M., Cooper M. (2024). Takotsubo cardiomyopathy after solid organ transplantation: A scoping review. Ann. Med. Surg..

[7] Matar A.J., Keiler J., Bolognese A.C., Cimeno A., Whitmore C., Wehrle C.J., Aucejo F., Fedorova E., Aufhauser D., Subramanian R. (2025). Takotsubo cardiomyopathy following liver transplantation: A multicenter cohort study. Liver Transpl..

[8] Eslami V., Nazari R., Senobari N., Alirezaei A., Akbari T., Nasrollahizadeh A., Ebrahimi P., Ahmed R., Khan S.Q., Shahid F. (2025). Stress-Induced Cardiomyopathy Following Kidney Transplant. JACC Case Rep..

[9] Moradkhani A., Avateffazeli M., Naghashzadeh F., Soleimani S., Ahmadi Z.H., Jahangiri Fard A., Homaee S., Varahram M., Hajiali M., Shafaghi S. (2026). Takotsubo Cardiomyopathy Secondary to Lung Transplantation Followed by Reduced Ejection Fraction Heart Failure: A Case Report. Clin. Case Rep..

[10] Covino S., Marotta L., Delle Femine F.C., D’Arienzo D., Liccardo B., Natale F., Cimmino G., Pezzullo E., Mattucci I., Caputo A. (2025). The Conundrum of Biventricular Takotsubo Syndrome After Heart Transplantation: A Case Report. Transpl. Proc..

[11] Souki F.G., Caram N., Fouda E., Nicolau-Raducu R. (2025). Stress Cardiomyopathy Following Multivisceral Transplantation: A Case Report. Cureus.

[12] Zhao H.-Q., Guo Z., Tong X., Li S., Wu G.-D., Yu L.-H., Li A., Hou Y.-C., Tang R. (2025). Takotsubo Cardiomyopathy After Orthotopic Liver Transplantation: A Case Series. Transpl. Proc..

[13] Boudart C., Assam O., Veliziotis I., Vander Kuylen M., Perrin L. (2024). Takotsubo syndrome-induced cardiogenic shock in lung transplantation: Importance of early diagnosis and ECMO implantation. Am. J. Case Rep..

[14] Shikama T., Shikama M., Hayase N., Doi K. (2025). Takotsubo syndrome following status epilepticus in a heart transplant recipient: A case report. Clin. Pract. Cases Emerg. Med..

[15] Yasumura K., Sera F., Akazawa Y., Ohtani T., Sakata Y. (2025). Three episodes of recurrent Takotsubo syndrome pre- and post-heart transplantation: A case report. Eur. Heart J. Case Rep..

[16] Pelliccia F., Kaski J.C., Crea F., Camici P.G. (2017). Pathophysiology of Takotsubo Syndrome. Circulation.

[17] Corruble E., Barry C., Varescon I., Durrbach A., Samuel D., Lang P., Castaing D., Charpentier B., Falissard B. (2011). Report of depressive symptoms on waiting list and mortality after liver and kidney transplantation: A prospective cohort study. BMC Psychiatry.

[18] Di Pietro G., Mennini G., Ciuffreda A., Birtolo L.I., Cimino S., Celli P., Ascione A., Iannello L., Galea N., Maestrini V. (2025). Reversible Tacrolimus-Induced Dilated Hypokinetic Cardiomyopathy. JACC Case Rep..

[19] Elezaby A., Dexheimer R., Sallam K. (2022). Cardiovascular effects of immunosuppression agents. Front. Cardiovasc. Med..

[20] Yang J., Rao Z., Hong F., Agopian V., Nguyen-Lee J., Steadman R.H., Wray C., Xia V.W. (2021). Takotsubo syndrome after liver transplantation: An association with intraoperatively administered epinephrine and fentanyl. Clin. Transpl..

[21] Zmaili M., Alzubi J., Alkhayyat M., Cohen J., Alkharabsheh S., Rana M., Alvarez P.A., Mansoor E., Xu B. (2022). Takotsubo cardiomyopathy in orthotopic liver transplant recipients: A cohort study using multi-center pooled electronic health record data. World J. Hepatol..

[22] Bachayev M., Brereton B., Mondal A., Alli-Ramsaroop B.A., Dhakal R., Leon M.C.B., Quinones C.M., Abdelal M.E.O., Jain A., Dhaduk K. (2024). Takotsubo Syndrome in Orthotopic Liver Transplant: A Systematic Review and Pooled Analysis of Published Studies and Case Reports. Transpl. Proc..

[23] Tachotti Pires L.J., Cardoso Curiati M.N., Vissoci Reiche F., Silvestre O.M., Mangini S., Carballo Afonso R., Ferraz-Neto B.H., Bacal F. (2012). Stress-induced cardiomyopathy (Takotsubo cardiomyopathy) after liver transplantation—Report of two cases. Transpl. Proc..

[24] Maestas C., Lazkani M., Sultan M., Kolli G., Sheikh M., Cherukuri M. (2019). Severe takotsubo cardiomyopathy following orthotopic liver transplantation: A case series. Clin. Res. Hepatol. Gastroenterol..

[25] Galván N.T., Kumm K., Kueht M., Ha C.P., Yoeli D., Cotton R.T., Rana A., O’Mahony C.A., Halff G., Goss J.A. (2018). Mending a Broken Heart: Treatment of Stress-Induced Heart Failure after Solid Organ Transplantation. J. Transpl..

[26] Vachiat A., McCutcheon K., Mahomed A., Schleicher G., Brand L., Botha J., Sussman M., Manga P. (2016). Takotsubo cardiomyopathy post liver transplantation. Cardiovasc. J. Afr..

[27] Chrapko B.E., Tomaszewski A., Jaroszyński A.J., Furmaga J., Wysokiński A., Rudzki S. (2012). Takotsubo syndrome in a patient after renal transplantation. Med. Sci. Monit..

[28] Gołębiewska J., Stopczyńska I., Dębska-Ślizień A., Bohdan M., Gruchała M., Rutkowski B. (2014). Tako-tsubo cardiomyopathy on the first day after renal transplantation—Case report and literature review. Transpl. Proc..

[29] Vailas M.G., Vernadakis S., Kakavia K., Paizis I., Bokos J., Boletis J., Zavos G. (2016). A heartbreaking renal transplantation: Is norepinephrine the culprit to blame?. Transpl. Proc..

[30] Michel-Cherqui M., Felten M.-L., Liu N., Sage E., Devaquet J., Grenet D., Fischler M. (2010). Management of Takotsubo Cardiomyopathy in a Lung Transplant Recipient. Transplantation.

[31] Ghadri J.R., Bataisou R.D., Diekmann J., Lüscher T.F., Templin C. (2015). First case of atypical takotsubo cardiomyopathy in a bilateral lung-transplanted patient due to acute respiratory failure. Eur. Heart J. Acute Cardiovasc. Care.

[32] Kato K., Cammann V.L., Napp L.C., Szawan K.A., Micek J., Dreiding S., Levinson R.A., Petkova V., Würdinger M., Patrascu A. (2021). Prognostic impact of acute pulmonary triggers in patients with Takotsubo syndrome: New insights from the International Takotsubo Registry. ESC Heart Fail..

[33] Omosule A., Malik M.F., Cisneros L., Guruswamy J. (2019). Takotsubo Cardiomyopathy After Double-Lung Transplantation: Role of Early Extracorporeal Membrane Oxygenation Support. J. Cardiothorac. Vasc. Anesth..

[34] Itzhaki Ben Zadok O., Ben-Avraham B., Hamdan A., Tovar A., Lyon A., Ben Gal T. (2020). A rare case of Takotsubo syndrome in a patient 5 months after heart transplantation. ESC Heart Fail..

[35] Gastwirth V.G., Yang H.S., Steidley D.E., Scott R.L., Chandrasekaran K. (2009). Dobutamine stress-induced cardiomyopathy in an orthotopic heart transplant patient. J. Heart Lung Transpl..

[36] Chinali M., Formigari R., Grutter G. (2018). Takotsubo cardiomyopathy in a young adult with transplanted heart: What happened to denervation?. ESC Heart Fail.

[37] Al Humaid M., Ahmed M., Galzerano D., Al Hussein M., Al Admawi M., Al Buraiki J., Alsergani H., Vriz O. (2019). A rare case of Takotsubo syndrome within the first day after heart transplantation. Monaldi Arch. Chest Dis..

[38] Miyake R., Ohtani K., Hashimoto T., Yada R., Sato T., Shojima Y., Hayashidani S., Higo T., Tsutsui H. (2020). Takotsubo syndrome in a heart transplant recipient with poor cardiac sympathetic reinnervation. ESC Heart Fail..

[39] Vailas M.G., Moris D., Papalampros A., Sotiropoulou M., Schizas D., Davakis S., Vernadakis S. (2017). A “Trapped Heart” in an Octopus Pot: Takotsubo Cardiomyopathy; Review of a Rare Clinical Syndrome Following Solid-Organ Transplant. Exp. Clin. Transpl..

[40] Buendia-Fuentes F., Almenar L., Ruiz C., Vercher J.L., Sanchez-Lazaro I., Martinez-Dolz L., Navarro J., Bello P., Salvador A. (2011). Sympathetic reinnervation 1 year after heart transplantation, assessed using iodine-123 metaiodobenzylguanidine imaging. Transpl. Proc..

[41] Kobashigawa J., Zuckermann A., Potena L., Ardehali A., Berman M., Chang P.P., Esmailian F., Farr M., Hall S., Hsich E. (2026). Summary of the International Society for Heart and Lung Transplantation (ISHLT) Consensus Conference on Graft Dysfunction within the First 72 hours after Heart Transplantation: A 10-year Update. J. Heart Lung Transpl..

[42] Velleca A., Shullo M.A., Dhital K., Azeka E., Colvin M., DePasquale E., Farrero M., García-Guereta L., Jamero G., Khush K. (2023). The International Society for Heart and Lung Transplantation (ISHLT) guidelines for the care of heart transplant recipients. J. Heart Lung Transpl..

[43] Berman M., Ali A., Ashley E., Freed D., Clarke K., Tsui S., Parameshwar J., Large S. (2010). Is stress cardiomyopathy the underlying cause of ventricular dysfunction associated with brain death?. J. Heart Lung Transpl..

[44] Giovannico L., Fischetti G., Mazzone F., Parigino D., Savino L., Paradiso I., Mezzina M., Urgesi E., Leo C., Cristiano G. (2026). Heart Transplantation from Donors with Takotsubo Cardiomyopathy: Clinical Outcomes and Early Experience from a Single Center. J. Clin. Med..

[45] Badwan O.Z., Abu-Shawer O., Paul A., Faulx M., Barzilai B. (2023). Postoperative apical ballooning syndrome following orthotopic liver transplantation. Cureus.

[46] Pal A., Malik A. (2024). Intraoperative Reverse Takotsubo Cardiomyopathy in Liver Transplantation: An Anesthesiologist’s Nightmare. Transpl. Proc..

[47] Jehangir M. (2025). Takotsubo cardiomyopathy after orthotopic liver transplantation. Int. J. Cardiovasc. Imaging.

[48] Ghadri J.R., Wittstein I.S., Prasad A., Sharkey S., Dote K., Akashi Y.J., Cammann V.L., Crea F., Galiuto L., Desmet W. (2018). International Expert Consensus Document on Takotsubo Syndrome (Part II): Diagnostic Workup, Outcome, and Management. Eur. Heart J..

[49] Yalta K., Madias J.E., Kounis N.G., Y-Hassan S., Polovina M., Altay S., Mebazaa A., Yilmaz M.B., Lopatin Y., Mamas M.A. (2024). Takotsubo Syndrome: An International Expert Consensus Report on Practical Challenges and Specific Conditions (Part-1: Diagnostic and Therapeutic Challenges). Balk. Med. J..

[50] Budnik M., Kochanowski J., Piatkowski R., Wojtera K., Peller M., Gaska M., Glowacka P., Karolczak P., Ochijewicz D., Opolski G. (2016). Simple markers can distinguish Takotsubo cardiomyopathy from ST segment elevation myocardial infarction. Int. J. Cardiol..

[51] Cammann V.L., Schweiger V., Szawan K.A., Di Vece D., Niederseer D., Würdinger M., Schönberger A., Schönberger M., Koleva I., Mercier J.C. (2026). Mechanical circulatory support for cardiogenic shock in takotsubo syndrome. Clin. Res. Cardiol..

[52] El-Battrawy I., Santoro F., Stiermaier T., Möller C., Guastafierro F., Novo G., Novo S., Mariano E., Romeo F., Romeo F. (2021). Incidence and Clinical Impact of Right Ventricular Involvement (Biventricular Ballooning) in Takotsubo Syndrome: Results From the GEIST Registry. Chest.

[53] Pelliccia F., Pasceri V., Patti G., Tanzilli G., Speciale G., Gaudio C., Camici P.G. (2019). Long-Term Prognosis and Outcome Predictors in Takotsubo Syndrome: A Systematic Review and Meta-Regression Study. JACC Heart Fail..

[54] von Mackensen J.K.R., Zwaans V.I.T., El Shazly A., Van Praet K.M., Heck R., Starck C.T., Schoenrath F., Potapov E.V., Kempfert J., Jacobs S. (2024). Mechanical Circulatory Support Strategies in Takotsubo Syndrome with Cardiogenic Shock: A Systematic Review. J. Clin. Med..

[55] Kosek-Nikołajczuk M., Borowiak E., Piatkowski R., Grabowski M., Budnik M. (2025). Long-Term Prognosis, Risk Assessment, and Management of Patients Diagnosed with Takotsubo Syndrome: A Narrative Review. J. Pers. Med..

