# Supplementary material for: Takotsubo Syndrome in Solid Organ Transplant Recipients: Current Evidence, Clinical Challenges, and Future Perspectives—A Narrative Review
Source: J Clin Med. 2026 Oct 20;15(20):8167. doi: 10.3390/jcm15208167 (PMC13705804; doi:10.3390/jcm15208167)
Supplement: Supplementary file 1 [file jcm-15-08167-s001.zip › jcm-4541470-supplementary.pdf]

**Supplementary Table S1.** PubMed search string			
Records retrieved	Restrictions	Publication cut-off	Database
88	English language	11 August 2026	PubMed
("Takotsubo syndrome"[Title/Abstract] OR "Takotsubo cardiomyopathy"[Title/Abstract] OR "stress cardiomyopathy"[Title/Abstract]) AND ("solid organ transplantation"[Title/Abstract] OR transplantation[Title/Abstract] OR "liver transplantation"[Title/Abstract] OR "kidney transplantation"[Title/Abstract] OR "renal transplantation"[Title/Abstract] OR "lung transplantation"[Title/Abstract] OR "heart transplantation"[Title/Abstract])			
